# What is the risk of dislocation after hip arthroplasty for patients with neuromuscular disease: a systematic review and meta-analysis

**DOI:** 10.1530/EOR-2025-0035

**Published:** 2026-06-01

**Authors:** Peng Wang, Qianqian Dai, Hanlong Xin, Shaohua Fan, Zhen Chen, Huitao Liu

**Affiliations:** ^1^ Department of Orthopedics, Taizhou Hospital of Zhejiang Province Affiliated to Wenzhou Medical University, Taizhou, Zhejiang, China; ^2^ Department of Emergency, Taizhou Hospital of Zhejiang Province Affiliated to Wenzhou Medical University, Taizhou, Zhejiang, China

**Keywords:** hip arthroplasty, neuromuscular disease, meta-analysis, dislocation

## Abstract

**Purpose:**

- Recent research has demonstrated excellent functional outcomes for patients with neuromuscular (NM) disease undergoing hip arthroplasty (HA). However, significant concerns persist regarding a potentially elevated risk of postoperative instability and dislocation in this specific cohort. Therefore, our objective was to synthesize the existing evidence through a systematic review and meta-analysis to evaluate the risks of dislocation in NM patients after HA. The purpose of this study was to answer the following question using a systematic review and meta-analysis: for patients undergoing HA (P), does the presence of a NM disease (I) increase the risk of postoperative dislocation (O) compared to patients without a NM disease (C)?

**Methods:**

- A meta-analysis was conducted from inception to December 2024, utilizing multiple database searches (PubMed, Embase, Web of Science, and Cochrane Library). Two authors independently selected studies, assessed their quality, and extracted relevant data. Disagreements were resolved through discussion with a third author. A fixed-effects meta-analysis model was used to calculate pooled odds ratios (ORs) with 95% confidence intervals (CIs). Statistical heterogeneity was assessed using the *I*^2^ statistic.

**Results:**

- Six studies were included, involving 22,803 patients; 2,643 NM and 20,160 non-NM underwent HA. We found the risk of dislocation in the NM group is higher than that in the non-NM group (OR: 1.59; 95% CI: 1.24-2.03; *I*^2^ = 0%); the NM group of elderly patients with combined femoral neck fractures also increases (OR: 1.63; 95% CI: 1.22–2.17; *I*^2^ = 0%).

**Conclusion:**

- In conclusion, the findings of this systematic review and meta-analysis provide robust evidence that patients with pre-existing NM disease face a substantially elevated risk of postoperative dislocation following HA. Our meta-analysis indicates that patients with NM diseases are at a heightened risk of dislocation following HA (OR: 1.59; 95% CI: 1.24-2.03, *P* < 0.001). This finding underscores the need for heightened preoperative counseling, meticulous surgical technique, and potentially the use of enhanced stability constructs in this vulnerable patient group.

## Introduction

Multiple patients suffering from neuromuscular (NM) disease benefited from hip arthroplasty (HA). The indications are three: primary osteoarthritis, secondary arthritis due to dysplasia, and femoral neck fractures. NM diseases can be divided into two categories: one accompanied by decreased muscle tone and the other related to increased muscle tone, contraction, or movement disorders. The former includes diseases such as polio and Down syndrome, while the latter includes diseases such as cerebral palsy (CP), Parkinson’s disease, and stroke (1).

CP patients often experience hip subluxation due to hip flexion and adduction contracture, aggravated hip valgus, and femoral forward tilt (2, 3). Sacroiliac joint deformity can lead to symptomatic joint disease, further limiting the ability to walk, stand, and sit (4). Conservative treatment of the hip joint may play a role in the early stages of the disease, but once joint lesions occur, severe hip joint lesions require surgical treatment to alleviate pain and optimize function (5, 6). Considering the good prognosis after HA surgery (7), the increase in hip osteoarthritis in the CP population (8), and the prolonged life expectancy of CP patients over time (9), HA is increasingly being considered for CP patients who have failed conservative measures (10).

Due to aging population and a high incidence of osteoporosis, hip fractures remain a major public health issue. Patients with NM diseases often struggle to maintain balance due to limb weakness and the loss of gross and fine motor abilities in the lower limbs. There is evidence to suggest that patients with NM diseases have an increased risk of falling (11, 12), and the intrinsic factors that contribute to this risk are far greater than the external factors (11). Patients with NM diseases have a significantly increased risk of osteoporosis, which may not only be related to inactivity. As the risk of falls and osteoporosis increases, the risk of hip fractures in patients with NM diseases is four times higher than that in normal individuals, with hemiplegic side fractures being the most common (13, 14). A study on 146 hemiplegic patients showed that 114 cases (82%) had hip fractures on the hemiplegic side, and 25 cases (18%) had hip fractures on the healthy side (15). It has been confirmed that joint replacement is the most cost-effective option for treating displaced femoral neck fractures in the elderly (16). The main advantage of joint replacement surgery for patients with displaced femoral neck fractures is to prevent fracture nonunion and bone necrosis. According to reports, this proportion is as high as 39% among patients receiving internal fixation treatment (17).

Recent studies have shown encouraging and positive outcomes for patients with CP undergoing HA, demonstrating significant pain relief and functional improvement (18, 19). Despite these benefits, there is a huge controversy in the clinic. Surgeons may be reluctant to offer HA to this group due, in part, to a well-documented high-risk profile of these patients, especially concerning postoperative instability and dislocation (20, 21, 22). This clinical equipoise highlights the need for a clear, evidence-based quantification of these risks.

## Methods

This meta-analysis adhered to the Preferred Reporting Items for Systematic Reviews and Meta-Analyses (PRISMA) guidelines (23). The protocol for this meta-analysis was registered in PROSPERO (Registration No. CRD42024538064).

### PICO question format

#### Population

Studies including adult patients undergoing primary HA were eligible.

#### Intervention/exposure

Included studies must have reported on a cohort of patients with a confirmed diagnosis of an NM (e.g. Parkinson’s disease, CP, and post-stroke hemiplegia).

#### Comparison

Studies were required to include a comparator or control group of patients undergoing HA who did not have an NM disease.

#### Outcome

The primary outcome of interest was the incidence of postoperative HA dislocations, reported separately for both the NM and the non-NM groups.

### Search strategy

Two authors searched the Cochrane Library, PubMed, Web of Science, and Embase databases for relevant research from the beginning until December 28, 2024. They retrieved the relevant studies using the following keywords: (neuromuscular disease [MESH]) OR (Neurological deficit [Title/Abstract]) OR (Hemiplegia [Title/Abstract]) OR (Neuromuscular imbalance [Title/Abstract]) OR (Neuromuscular conditions [Title/Abstract]) OR (Neuromuscular disorder [Title/Abstract]) OR (Cerebral palsy [Title/Abstract]) OR (Paralysis [Title/Abstract]) AND (Arthroplasty [MESH]) OR (Joint replacement [Title/Abstract]) OR (Total hip arthroplasty [Title/Abstract]) OR (THA [Title/Abstract]) OR (Total joint replacement [Title/Abstract]) OR (TJR [Title/Abstract]) OR (Hemiarthroplasty [Title/Abstract]) OR (HA [Title/Abstract]). All studies that met the search criteria were reviewed, regardless of the main results and language.

### Eligibility criteria and data extraction

This evaluation criteria was agreed in advance, and two researchers screened the retrieved literature based on inclusion and exclusion criteria and conducted independent evaluations. Any difference swere resolved through consensus with a third researcher. The conditions for inclusion in the study were randomized controlled trials, retrospective or prospective cohort studies, and case–control studies. The study population included patients with NM diseases, CP, and joint replacement surgery. We excluded studies without full text or original research data. The excluded research types include animal experiments, case reports, abstracts, editorial comments, letters, conference papers, reviews, and meta-analyses. The researchers excluded studies with duplicate publications from different databases. The extracted data included the name of the first author, publication year, country, general characteristics of the patient and control group (age and gender ratio), study and follow-up time, and results of long-term complications. The main outcome was the incidence of postoperative joint dislocation. The relevant systematic research and reference materials for the identified articles have also been reviewed to identify other studies that met the criteria.

### Quality assessment

Two authors independently used the Newcastle–Ottawa scale (NOS) to evaluate the quality of the included studies. Any differences will be resolved through discussions with a third researcher. NOS needs to evaluate three aspects (selection of study subjects, intergroup comparability, and evaluation of outcome indicators) to evaluate the quality of cohort studies. A high-quality research result requires a score of seven or more (24).

### Statistical analysis

All statistical analyses were conducted using Review Manager 5.3 (The Nordic Cochrane Centre, The Cochrane Collaboration 2014) within a frequentist framework. The primary summary statistic for dichotomous outcomes was the pooled odds ratio (OR) with its corresponding 95% confidence interval (CI). Statistical significance was set at a *P*-value less than 0.05.

The decision to use a fixed-effect or random-effects model was made *a priori* based on the *I*^2^ statistic to assess the degree of statistical heterogeneity. *I*^2^ value of 0% indicating no heterogeneity, and >50% was considered substantial heterogeneity. With respect to our primary outcomes, the analysis indicated no statistical heterogeneity (*I*^2^ = 0%) between the studies incorporated.

Thus, fixed effect, weighted by inverse variance, was applied to the final data analysis. We looked at recent advice on estimators and adjustments for heterogeneity. Recent methodological reviews indicate that the DerSimonian–Laird (DL) estimator commonly used in random effects models is unreliable and that more robust estimators with the Hartung–Knapp (HK) adjustment are best practice to avoid false positives (25). Nonetheless, the HK adjustment is designed for use in random-effects models and addresses uncertainty about variation between studies. According to our investigation, the random-effects model was mathematically equivalent to the fixed-effect model, with an *I*^2^ of 0%, meaning the estimated between-study variance was zero. Consequently, our final model did not apply the HK adjustment. A funnel plot was created to evaluate the possible publication bias visually.

A funnel plot was generated to visually assess for potential publication bias. However, it is critical to note that with a limited number of included studies (*n* < 10), the power of funnel plots to detect true bias is low, and any observed asymmetry should be interpreted with extreme caution. Asymmetry in this context can easily arise from chance or between-study heterogeneity rather than publication bias, and therefore, no definitive conclusions can be drawn from visual inspection alone. Formal statistical testing, such as Egger’s test, was not performed due to being underpowered and unreliable with fewer than 10 studies.

## Results

### Study characteristics

**Figure 1:**
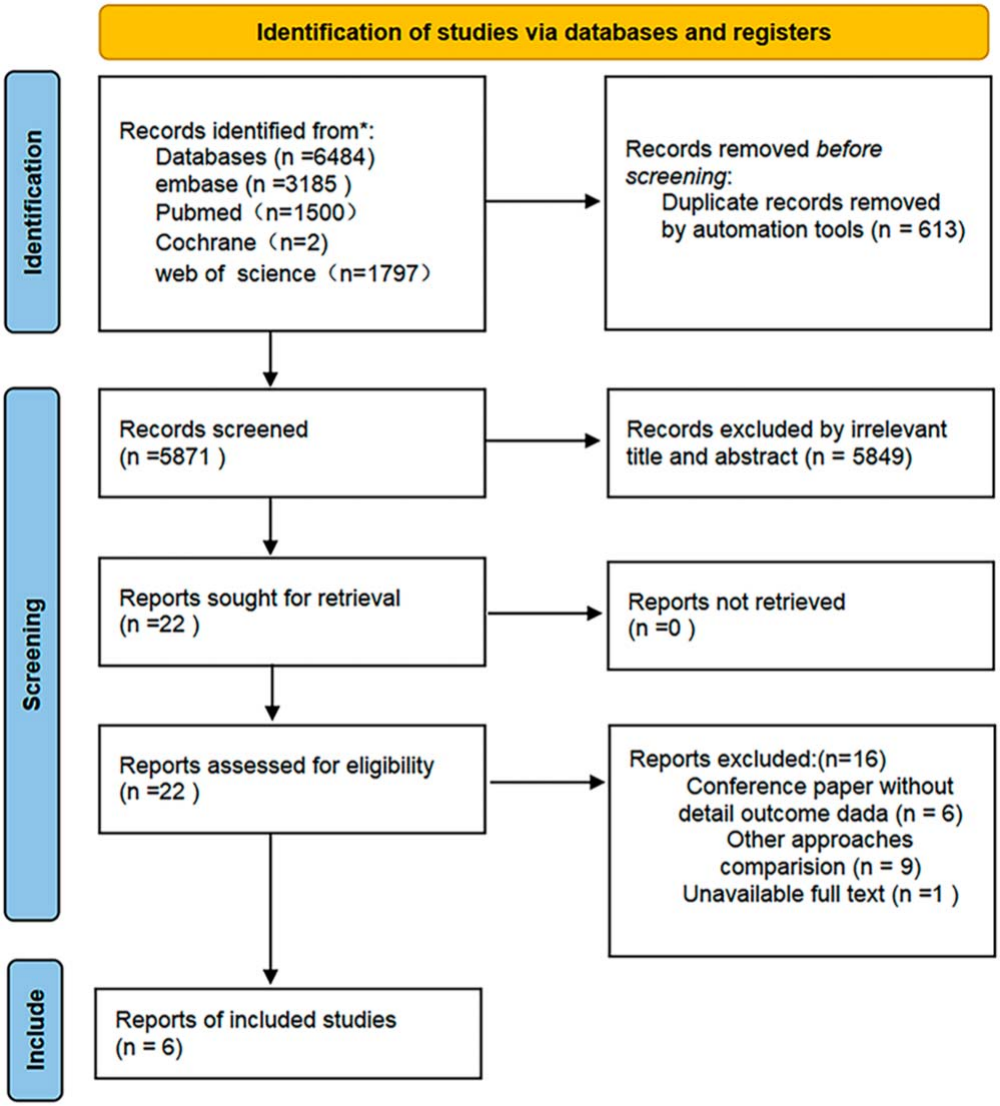
Flow chart of search and selection for included studies.

According to the PRISMA guidelines, the flow chart illustrating the selection process for the included studies is presented in Fig. 1. A total of 6,484 related studies were identified from the database based on the retrieval strategy (Web of Science = 1,797, Embase = 3,185, PubMed = 1,500, and Cochrane Library = 2). EndNote 8X (Thomson Reuters Corp, USA) software was used to remove duplicate publications, leaving 5,781 studies remaining. After filtering the titles and abstracts, 5,849 studies were deleted. The full text of the remaining 22 studies was read, and 16 more studies were excluded. Ultimately, 6 studies that met the inclusion criteria were included in the meta-analysis (Fig. 1), with 22,803 patients (NM group = 2,643 and non-NM group = 20,160). The basic characteristics of the included studies are summarized in Table 1 (26, 27, 28, 29, 30, 31). The publication period was from 2012 to 2023, and the follow-up period ranged from 12 to 240 months. The quality of the included studies was evaluated using the NOS scale, and the NOS scores of all studies indicated that they were of high quality (Table 1).

**Table 1:** General characteristics of included studies.

Study	Year	Study period	Country	Study design	Sample size (NM/non-NM)	Female (NM/non-NM)	NM age^†^, years	FU, months	Dislocation (NM/non-NM)	NOS score^a^		
										S	C	O
Moore *et al.* (26)	2021	2010–2018	USA	RS	864/3,448	465/1,856	56.3 ± 13.5	60	23/63	****	**	***
Ryu *et al.* (27)	2021	2013–2015	Korea	PS	35/127	27/87	77.6 ± 8.4	60–88	2/5	****	**	***
Suh *et al.* (28)	2012	1996–2008	Korea	RS	42/148	25/97	75.3 ± 8.5	13–76	2/3	****	**	***
Awadallah *et al.* (29)	2022	1986–2020	UK	RS	323/3,371	217/2,662	80.1 ± 8.1	12	8/38	****	*	***
Houdek *et al.* (30)	2017	1990–2013	USA	RS	39/78	13/26	48.7 ± 12.3	24–240	3/3	****	**	***
Zhang *et al.* (31)	2023	2010–2020	USA	RS	1,340/12,988	773/7,558	76.4 ± 3.6	24–108	44/281	****	**	***

RS, retrospective study; PS, prospective study; NM, neuromuscular disease; non-NM, non-neuromuscular disease; FU, follow-up.

Score^a^: (S) selection of study groups; (C) comparability; (O) outcome assessment.

^†^ Values are mean ± SD.

### Risk of bias

Considering the small sample size of our meta-analysis (<10), publication bias is not applicable to this study.

### Population characteristics

We collected data from 22,803 patients. There were a total of 2,643 cases in the NM group, with an average of 57.5% being females. There were a total of 20,160 patients in the non-NM group, of which 60.9% were females. We have summarized the demographic data of patients (Table 2).

**Table 2:** Demographic data of the included studies.

Study	Hips (*n*)	NM group			Non-NM group		
		Patients (*n*)	Female (%)	Mean age	Patients (*n*)	Female (%)	Mean age
Moore *et al.* (26)	4,312	864	53.8	56.3 ± 13.5	3,448	53.8	56.0 ± 13.6
Ryu *et al.* (27)	162	35	77.1	77.6 ± 8.4	127	68.5	76.2 ± 7.9
Suh *et al.* (28)	190	42	59.5	75.3 ± 8.5	148	65.5	76.8 ± 6.6
Awadallah *et al.* (29)	3,694	323	67.2	80.1 ± 8.1	3,371	78.9	83.0 ± 8.6
Houdek *et al.* (30)	117	39	33.3	48.7 ± 12.3	78	33.3	48.8 ± 11.0
Zhang *et al.* (31)	14,328	1,340	57.7	76.4 ± 3.6	12,988	58.2	76.5 ± 3.1

**Figure 2:**
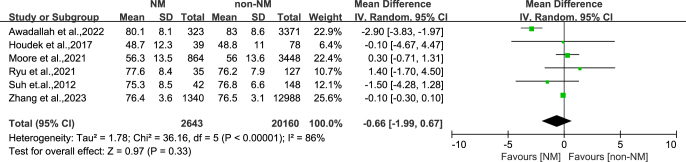
Subgroup analysis of age.

**Figure 3:**
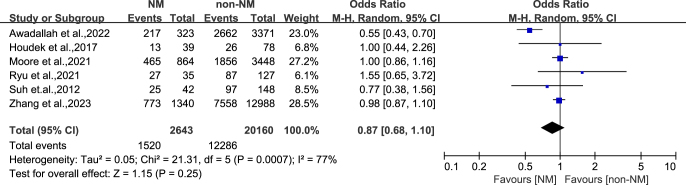
Subgroup analysis of gender.

There was no statistically significant difference in the age of patients included in the NM group compared to the non-NM group (OR: -0.66, 95% CI: -1.99-0.67, *P* = 0.33) (Fig. 2). Compared with the non-NM group, the gender of patients included in the NM group (OR: 0.87, 95% CI: 0.68–1.10, *P* = 0.25) showed no statistically significant difference between the two groups (Fig. 3). This indicates that age and gender are likely to be important confounding factors for differences in outcomes between the two groups. Other important confounding factors, such as weight, were not considered in individual studies. Further analysis is not possible.

### Dislocation

**Figure 4:**
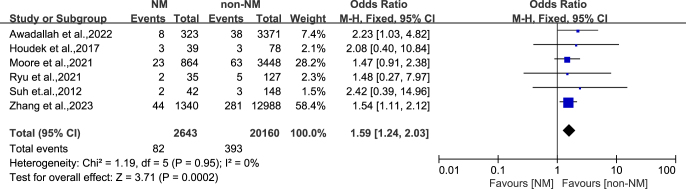
Subgroup analysis of the risk of hip dislocation.

**Figure 5:**
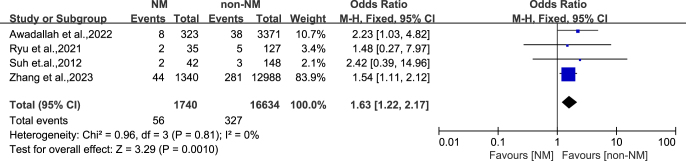
Subgroup analysis of the risk of hip dislocation with combined femoral neck fractures.

Quantitative analysis of hip joint dislocation is shown in Figs. 4 and 5. Six studies reported the risk of postoperative dislocation in 22,803 HA patients (2,643 in the NM group and 20,160 in the non-NM group). By summarizing the data on postoperative dislocation risk, it was found that the dislocation rate (OR: 1.59, 95% CI: 1.24-2.03, *P* < 0.001) in the NM group was consistently higher than that in the non-NM hip replacement group (Fig. 4). Four studies reported the risk of postoperative dislocation in 18,374 elderly patients with femoral neck fractures who underwent HA surgery (1,740 in the NM group and 16,634 in the non-NM group). By summarizing these data, it was found that the dislocation rate (OR: 1.63, 95% CI: 1.22–2.17, *P* = 0.001) of elderly patients with NM combined with femoral neck fractures was significantly higher than that of non-NM hip replacement patients (Fig. 5).

### Publication bias

**Figure 6:**
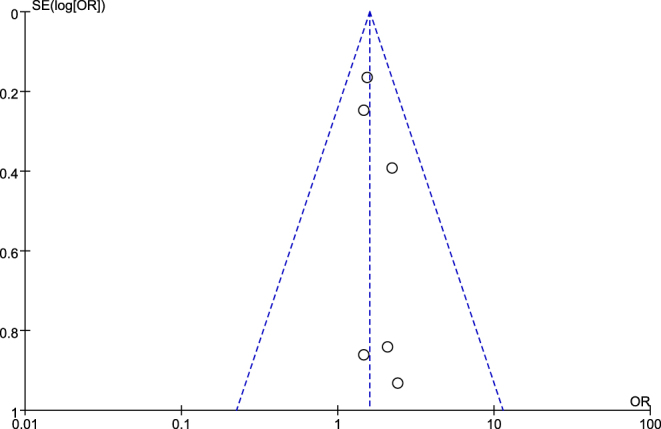
Funnel plots of the risk of hip dislocation.

**Figure 7:**
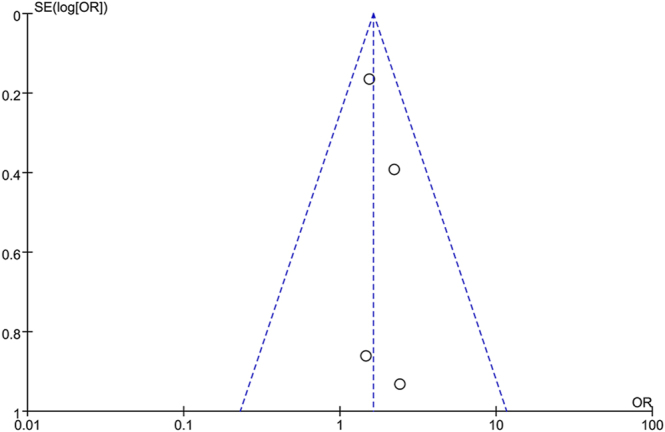
Funnel plots of the risk of hip dislocation with combined femoral neck fractures.

The creation of a funnel plot, aiming to determine whether publication bias exists for the dislocation rate, was an aspect of this review (Figs. 6 and 7). The plot displays the effect estimates from the individual studies against their precision. The studies may be symmetrically distributed about the summary effect estimate in a visual sense. In our methods, we stated that if only six or four studies are included, it cannot be interpreted. Based on this visual, it is not possible to rule out or in publication bias with any confidence.

## Discussion

The primary finding of this systematic review and meta-analysis is that patients with a pre-existing NM disease have a statistically significant and clinically meaningful increased risk of dislocation following HA. Our pooled analysis quantifies this risk, demonstrating that the odds of postoperative dislocation are 59% higher in the NM disease cohort compared to patients without comorbidities (OR: 1.59, 95% CI: 1.24-2.03, *P* = 0.0002). This result provides robust, synthesized evidence to support the long-held clinical concern that NM disease is a major independent risk factor for instability after HA, a finding that holds true across different indications for surgery, from osteoarthritis in CP patients to femoral neck fractures in elderly patients with paralysis.

Our quantitative finding is consistent with the conclusions of several previous studies that have qualitatively associated NM conditions with a higher risk of dislocation (29, 32, 33). As per the reports, instances of dislocation after hemiarthroplasty or HA in the general population are quite rare, with a range of 0.8–5.0% (34); however, our study suggests that the presence of an NM disease significantly increases this baseline risk. This is probably due to several contributing factors. As described in the literature, instability may occur due to soft tissue imbalance, muscle weakness, increased tone (e.g. Parkinson’s disease), and cognitive impairments interfering with compliance with postoperative precautions (33, 35, 36). We used meta-analysis to pull together these individual observations to give one overall effect size.

The literature on specific NM diseases, such as CP, shows evidence that the clinical dilemma has been highlighted in our findings. There has been a long-standing reluctance among surgeons to perform HA for CP patients due to fears of complications such as dislocation and early failure of the prosthesis (30, 37). Multiple studies show HA is effective for pain relief and functional improvement in these patients (22, 37), but the risk profile remains a concern. The literature also features a debate regarding the longevity of implants in CP patients. Some studies report that the 10-year implant survival rate has been excellent at around 85% (18), as presented in our included studies. In contrast, other larger registry studies, King *et al.*, noted that implant survival at 5 years was significantly worse in CP patients. This may be due to the greater preoperative deformity leading to a greater incidence of periprosthetic fractures (38). Our findings not only help to quantify instability as one parameter of risk but also highlight the complexity involved in treating such patients.

A systematic and meta-analytic approach is the main strength of this study. The pooling of data from six studies comprising 22,803 patients results in greater power and a more exact risk estimation than any one study could offer. This broad perspective aids in bringing together a disconnected array of information into a single conclusion. Nonetheless, the study had limitations as well. To begin, both prospective and retrospective studies were among the included evidence, while there was variability in the methodological quality. A limitation identified from this review was the small number of high-quality studies, which prevented the possibility of performing subgroup analyses for certain NM diseases (e.g. CP vs muscular dystrophy vs post-stroke). In the next order of finding, there was variability in terms of study design, NM disease definitions, and follow-up periods. These factors may have contributed to statistical heterogeneity. In the end, the deficiency of more detailed and longitudinal information related to dislocation charges and functional outcomes in distinct NM disease populations is a major gap in the literature.

Our findings have clinical implications that are direct and actionable. The quantified 59% increase in the odds of dislocation should be a key component of preoperative counseling and shared decision-making for HA in patients with NM diseases. This evidence provides a compelling rationale for surgeons to employ special considerations for this high-risk group. This includes meticulous surgical technique focused on prosthetic positioning, optimizing offset and version, and careful soft tissue tensioning and repair (39). Moreover, this may justify the preferential use of increased stability constructs, such as dual-mobility cups or larger femoral heads, because of the inherent risk. These patients may benefit from individualized rehabilitation schemes and further measures to prevent dislocation. Looking forward, there is a clear need for future research. High-quality, prospective multicenter studies are urgently required to disaggregate the risk of dislocation and other complications by specific NM disease subtypes. Such studies would allow for the development of disease-specific treatment algorithms and provide the necessary evidence to conduct comparative effectiveness research on which implant constructs and surgical techniques yield the best long-term, stable outcomes in this vulnerable patient population.

## Conclusion

Based on our retrospective meta-analysis of existing studies, we found that patients with NM diseases have a higher risk of dislocation. Orthopedic doctors should be aware of these risks and provide a basis for the selection of surgical methods for NM patients. If joint replacement surgery is required, special attention should be paid to postoperative rehabilitation exercise guidance to reduce the occurrence of postoperative dislocation. However, this study is mainly a retrospective analysis; therefore, further prospective cohort studies are needed to confirm our results, although our meta-analysis provides important insights into the current literature on this topic.

## ICMJE Statement of Interest

The authors declare that there is no conflict of interest that could be seen as damaging the impartiality of the reported research.

## Funding Statement

This study was fully funded by internal sources and did not involve any commercial entities. The funding source did not participate in the design, data collection, data analysis, data interpretation, or report writing of the study. The corresponding author has full access to all research data and is ultimately responsible for deciding to submit it for publication.

## Author contribution statement

PW conceived the study. HL developed the research protocol. QD and SF performed the literature search. QD and HX screened titles and abstracts and reviewed full texts. PW and ZC performed data abstraction. PW submitted the review to PROSPERO. PW prepared the first manuscript draft. All authors contributed to final edits and revisions prior to submission.
